# The XN-30 hematology analyzer for rapid sensitive detection of malaria: a diagnostic accuracy study

**DOI:** 10.1186/s12916-019-1334-5

**Published:** 2019-05-31

**Authors:** Annelies Post, Berenger Kaboré, Isaie J. Reuling, Joel Bognini, Wouter van der Heijden, Salou Diallo, Palpouguini Lompo, Basile Kam, Natacha Herssens, Kjerstin Lanke, Teun Bousema, Robert W. Sauerwein, Halidou Tinto, Jan Jacobs, Quirijn de Mast, Andre J. van der Ven

**Affiliations:** 1 Nijmegen Institute of International Health, Radboud University Medical Centre, Nijmegen, the Netherlands; 2 IRSS/Clinical Research Unit of Nanoro (CRUN), Nanoro, Burkina Faso; 3 Department of Medical Microbiology, Radboud University Medical Centre, Nijmegen, the Netherlands; 4 Department of Clinical Sciences, Institute of Tropical Medicine (ITM), Antwerp, Belgium; 5 Centre Muraz, Bobo-Dioulasso, Burkina Faso; 6 Institut Supérieur des Sciences de la Santé, Université Nazi Boni de Bobo-Dioulasso, Bobo-Dioulasso, Burkina Faso; 7 Department of Microbiology and Immunology, University of Leuven (KU Leuven), Leuven, Belgium

**Keywords:** Malaria, Diagnosis, Sensitivity, Specificity, Burkina Faso

## Abstract

**Background:**

Accurate and timely diagnosis of malaria is essential for disease management and surveillance. Thin and thick blood smear microscopy and malaria rapid diagnostic tests (RDTs) are standard malaria diagnostics, but both methods have limitations. The novel automated hematology analyzer XN-30 provides standard complete blood counts (CBC) as well as quantification of malaria parasitemia at the price of a CBC. This study assessed the accuracy of XN-30 for malaria detection in a controlled human malaria infection (CHMI) study and a phase 3 diagnostic accuracy study in Burkina Faso.

**Methods:**

Sixteen healthy, malaria-naive CHMI participants were challenged with five *Plasmodium falciparum-*infected mosquitoes. Blood was sampled daily for XN-30, blood smear microscopy, and malaria qPCR. The accuracy study included patients aged > 3 months presenting with acute febrile illness. XN-30, microscopy, and rapid diagnostic tests (HRP-2/pLDH) were performed on site; qPCR was done in retrospect. The malaria reference standard was microscopy, and results were corrected for sub-microscopic cases.

**Results:**

All CHMI participants became parasitemic by qPCR and XN-30 with a strong correlation for parasite density (*R*^2^ = 0.91; *p* < .0001). The XN-30 accurately monitored treatment and allowed detection of recrudescence. Out of 908 patients in the accuracy study, 241 had microscopic malaria (density 24–491,802 parasites/μL). The sensitivity and specificity of XN-30 compared to microscopy were 98.7% and 99.4% (PPV = 98.7%, NPV = 99.4%). Results were corrected for qPCR-confirmed sub-microscopic cases. Three microscopy-confirmed cases were not detected by XN-30. However, XN-30 detected 19/134 (14.2%) qPCR-confirmed cases missed by microscopy. Among qPCR-confirmed cases, XN-30 had a higher sensitivity (70.9% versus 66.4%; *p* = .0009) and similar specificity (99.6% versus 100%; *p* = .5) as microscopy. The accuracy of XN-30 for microscopic malaria was equal to or higher than HRP-2 and pLDH RDTs, respectively.

**Conclusions:**

The XN-30 is a novel, automated hematology analyzer that combines standard hemocytometry with rapid, objective, and robust malaria detection and quantification, ensuring prompt treatment of malaria and malaria anemia and follow-up of treatment response.

**Trial registration:**

Both trials were registered on clinicaltrials.gov with respective identifiers NCT02836002 (CHMI trial) and NCT02669823 (diagnostic accuracy study).

**Electronic supplementary material:**

The online version of this article (10.1186/s12916-019-1334-5) contains supplementary material, which is available to authorized users.

## Background

Malaria remains a major cause of morbidity and mortality around the world. Sub-Saharan Africa is most affected with *Plasmodium falciparum* accounting for 99% of estimated cases [1]. Timely and accurate diagnosis of malaria is essential for disease management and control [2]. Thin and thick blood smear microscopy (further referred to as microscopy) and malaria rapid diagnostic tests (RDTs) are standard malaria diagnostics in endemic areas. RDTs have significantly improved the use of diagnostics for malaria diagnosis, accounting for 74% of diagnostic tests performed among suspected cases in 2015 [3]. Both techniques provide challenges in clinical practice: RDTs are antigen-directed and relative to their design, can neither quantify malaria parasitemia, nor allow monitoring of treatment response, prerequisites to manage severe malaria. Additionally, RDTs cannot distinguish current from recently treated infections [4]. Consequently, false-positive results may be misinterpreted as treatment failure [5]. WHO therefore reserves a role for microscopy, which is however labor-intensive and its quality is heavily observer-dependent. Molecular tests are complex to perform, require highly trained personnel, and are relatively expensive, limiting its general use.

The XN-30 (Sysmex, Kobe, Japan) is a novel automated hematology analyzer and malaria diagnostic that directly detects and quantifies *Plasmodium* parasites (*falciparum* and non-*falciparum*) in blood using violet laser technology [6–8]. Our study aimed to evaluate the accuracy of the XN-30 for the detection of *Plasmodium falciparum* malaria. First, we assessed its performance in healthy participants in whom low-density parasitemia was induced in a controlled human malaria infection (CHMI) study. To establish the performance under field conditions, we then performed a phase 3 diagnostic accuracy study among febrile patients in Burkina Faso.

## Methods

### Controlled human malaria infection (CHMI)

The ability of the XN-30 to detect low-density sexual and asexual parasitemia and monitoring of treatment was studied in a CHMI trial (ClinicalTrials.gov, NCT02836002). Details and results of the primary objectives of this study have recently been published [9]. Ethylenediaminetetraacetic acid (EDTA)-anticoagulated venous blood was collected twice daily for XN-30, and results were compared with qPCR for asexual- and qRT-PCR for sexual *P. falciparum* parasites. Both techniques are described in detail elsewhere [9].

### Diagnostic accuracy study

This prospective, double-blinded, phase 3 diagnostic accuracy study (ClinicalTrials.gov, NCT02669823) was performed at the Clinical Research Unit of Nanoro (CRUN) [10]. The primary objectives were (i) to assess the diagnostic sensitivity and specificity of the XN-30 to detect malaria parasitemia in children and adults with an acute febrile illness against thick blood smear microscopy or qPCR in case of a negative thick smear or incongruent results of XN-30 and microscopy and (ii) to assess the diagnostic accuracy of the Infection Manager System (IMS) for the detection of bacterial bloodstream infection in a malaria-endemic area. The secondary objective was to compare the accuracy of the XN-30 analyzer to diagnose malaria compared to malaria RDT. The results of the IMS are reported elsewhere to increase legibility.

### Procedures

Nanoro is a rural area of Burkina Faso which is hyperendemic for *Plasmodium falciparum*, though *Plasmodium ovale* and *Plasmodium malariae* are sporadically found [11]. Participants were enrolled between March 2016 and June 2017 at the “Centre Medicale avec Antenne Chirurgicale” (CMA) of Nanoro, to which CRUN is affiliated. Consecutive patients of 3 months and older suspected of acute febrile illness were screened for eligibility. Patients were eligible if they had a measured temperature of ≥ 38.0 °C or ≤ 35.5 °C, or a reported history of fever up to 48 h prior to presentation, or suspicion of severe infection with signs of severe clinical illness. Patients with fever lasting more than 7 days were excluded. Upon inclusion, 2–5 mL EDTA anti-coagulated blood was obtained and analyzed within 1 h after sampling. Laboratory analyses were performed and interpreted by experienced laboratory technicians who were blinded to clinical data. Patients were followed daily during hospitalization, and follow-up samples were taken if clinically indicated. Another follow-up sample was taken at approximately 2 weeks after inclusion.

Data was collected on case report forms (CRFs) and entered into a secure database (RedCap, Vanderbilt University, Nashville, USA) after conformity check by a medical doctor. Entered data were checked against the CRFs by a data manager. Approximately 10 % of participant study files were checked by an independent monitor. Results from qPCR were entered into an Excel (Microsoft, Washington, USA) database and merged with the principal database. The researchers were blinded to the XN-30 until the clinical database was locked. Interpretation of the index test was done by blinded researchers after inclusion was completed. Laboratory procedures were standardized; quality controls were performed according to good clinical and laboratory practices (GCLP) guidelines.

### Index test: XN-30

**Fig. 1:**
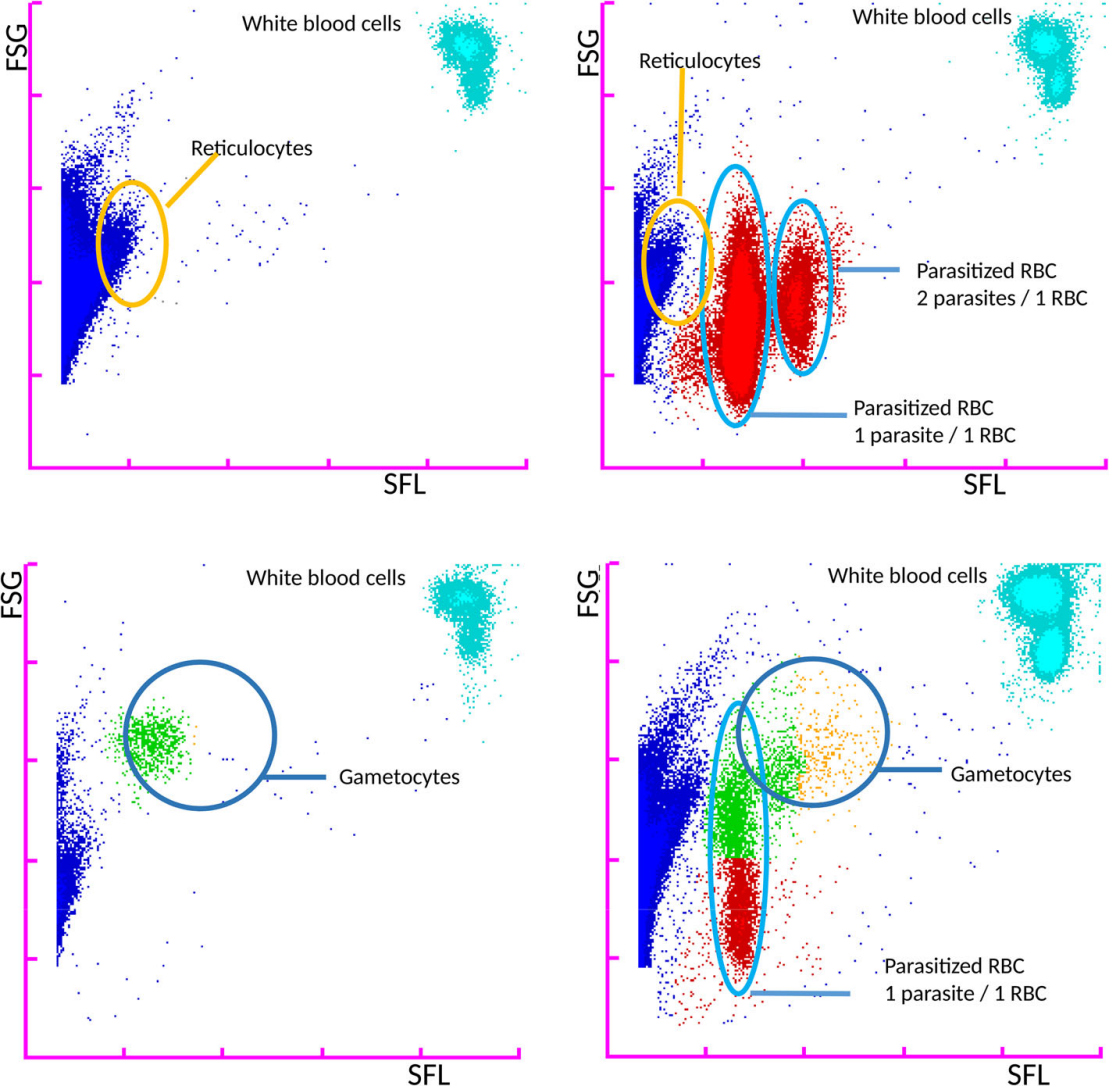
Forward sideward scatter of patient with and without malaria and gametocytes as recorded by the XN-30

The XN-30 is an automated hematology analyzer (Sysmex Corporation, Kobe, Japan) which can be used for malaria detection as described in detail elsewhere [6]. The analyzer aspirates and dilutes blood samples in a diluent solution (CELLPACK DCL). Subsequently, the nucleic acids are stained with a staining solution (Fluorocell M) along with a lysis solution (LysercellM). Infected red blood cells (iRBC) and white blood cells (WBC) are detected by a violet semiconductor 405 nm laser beam. Parasitemia percentage is calculated by the ratio of infected- and uninfected red blood cells. Output data separately reports a complete blood count (CBC), the presence of gametocytes and parasitemia—both as a percentage of infected red blood cells (MI-RBC%) and absolute parasite density (MI-RBC#) expressed as parasites/μL. The data is automatically determined by analyzing scatter grams plotted three dimensionally with forward-side scattered light, side scattered light, and side fluorescent light. iRBC are visible as a separate RBC population on the scattergram (Fig. 1). In case of abnormal scatter gram distribution, the malaria result is reported as “inconclusive.”

The analyzer aspirates approximately 60 μL whole blood per analysis; results are available within 1 min. XN-30 will be made for global distribution, and the price of a measurement is expected to be comparable to regular hemocytometry [6]). No formal quantification limit was applied for XN-30 for the CHMI study. In the diagnostic accuracy study, XN-30 values of ≥ 20 MI-RBC/μL with at least 5% ring stages were considered positive. Validated hemocytometry data were used for clinical care, all experimental data were kept blinded.

### Reference tests: malaria diagnostics

Both RDT and microscopy were performed using EDTA-anticoagulated blood. Thick and thin blood films were examined for the presence of *Plasmodium* parasites according to WHO procedures [12]. Results were expressed as asexual parasites per microliter using the patients’ WBC count. The *Plasmodium* species and presence of gametocytes were also recorded. Slides were viewed by two independent microscopists. Presence of one or more *Plasmodium* parasites was considered positive. In case of discrepancies, the result of a third microscopist was decisive.

SD Bioline Malaria Ag-P.f (HRP2/pLDH) (Standard Diagnostics, Inc., Gyeonggi-do, Republic of Korea) was performed according to the manufacturer’s instructions. Five microliters of venous blood was inoculated on the RDT cassette. After 5 and 15 min, the test line reactivity of the *Plasmodium falciparum* histidine-rich protein-2 (HRP-2) and pan-*Plasmodium* species parasite-lactate dehydrogenase (pLDH) were scored, whereby any visible test line was considered positive. Results for pLDH and HRP-2 were recorded separately. Quantitative PCR (qPCR) targeting the multicopy 18S rRNA gene was performed in retrospect at the Department of Microbiology of Radboud University Medical Centre, the Netherlands, using 200 μL blood per patient using previously described methods [13]. The lower level of detection of qPCR was 0.05 parasites per microliter (p/μL).

### Case definitions, data management, and statistical analysis

Malaria parasitemia was defined as the presence of one or more parasites in the asexual stage in malaria microscopy or a malaria qPCR result over 0.05 p/μL in the diagnostic accuracy study. In case only gametocytes were observed, microscopy was considered negative. Severe malaria was defined according to WHO criteria 2014 [14].

A statistical plan was made before data inspection. Analyses were done using Stata 14 (Stata Corp, College Station, TX, USA). Patients with missing data on either reference test, index test, or qPCR were excluded from the analysis. In the case of inconclusive index test results due to an abnormal scattergram distribution, the result was reported as “inconclusive.” Differences in proportions, medians, or means were compared using respectively the chi-square test, two-tailed Fisher’s exact test, or Mann-Whitney *U* test in case of not normally distributed data. Sensitivity, specificity, positive predictive value (PPV), and negative predictive value (NPV) were assessed using the STATA diagt-package. A receiver operation characteristics (ROC) curve analysis was done to assess the area under the curve (AUC). Linear regression analyses were done to assess the correlation in parasite densities between qPCR, malaria thick smear, and XN-30. Comparative analyses of sensitivity and specificity were done using a McNemar test and reported as test ratio with significance level. A significance level of 5% was used for all analyses.

## Results

### Controlled human malaria infection

**Fig. 2:**
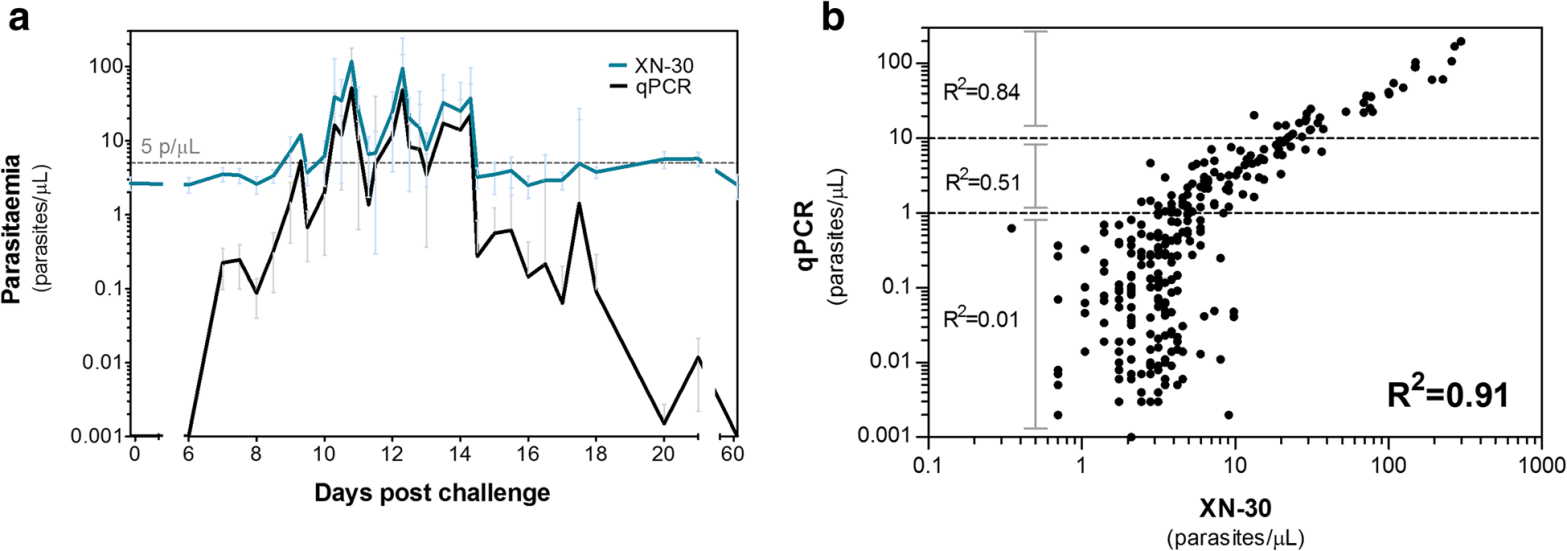
Diagnostic performance of XN-30 in CHMI model expressed as (**a**) line graph and (**b**) scatterplot

**Fig. 3:**
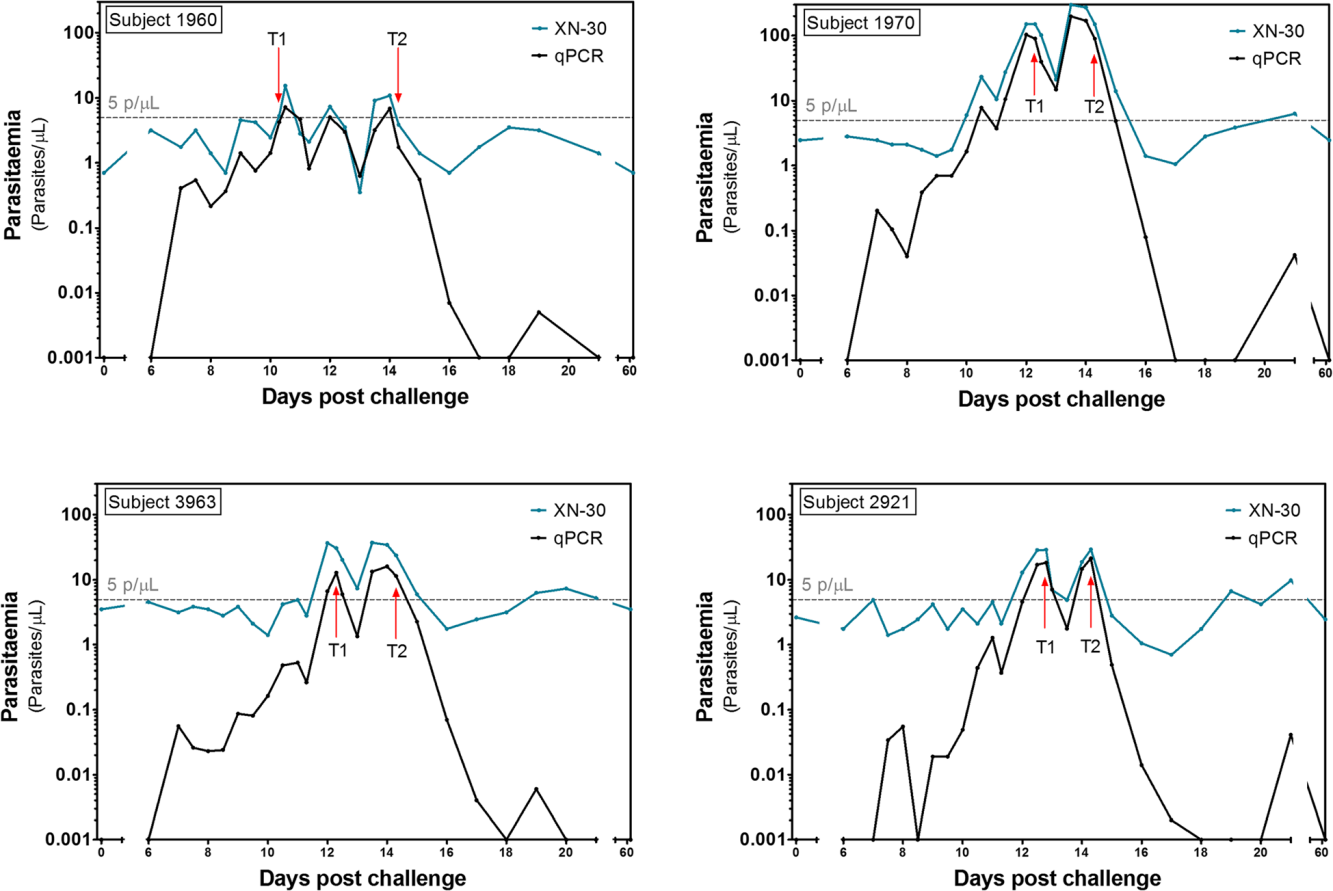
Parasite density curves of participants with recrudescent infections. The parasite density treatment threshold of 5 p/uL is indicated with a grey line

We assessed the diagnostic accuracy of XN-30 in 16 healthy participants exposed to low-density parasitemia during a CHMI (Additional file 3: Figure S1). All participants developed parasitemia between day 6 and 12 post-infection, as determined by qPCR. The geometric mean peak parasitemia density was 21 p/μL. Figure 2a shows parasitemia dynamics over time as quantified by the XN-30 analyzer and by qPCR. The quantitative data shows a strong correlation between the XN-30 and qPCR (*R*^2^ = 0.91; *p* < .0001) (Fig. 2b). The XN-30 was able to monitor treatment and detect recrudescent infections (Fig. 3). The positive cutoff of the XN-30 was 8.1 p/μL as defined by the mean of all negative qPCR samples + 3 SD. Gametocyte densities in CHMI were too low to be detected by microscopy or XN-30 (< 1.3 gametocytes/μL). In vitro experiments were performed to estimate the limit of detection of mature gametocytes and test the stability of the samples when measured by XN-30 (Additional file 4: Figure S2 and Additional file 6: Figure S4). Sample quantification remained accurate up to 6 h after sampling, at room temperature (Additional file 5: Figure S3).

### Diagnostic accuracy study

**Fig. 4:**
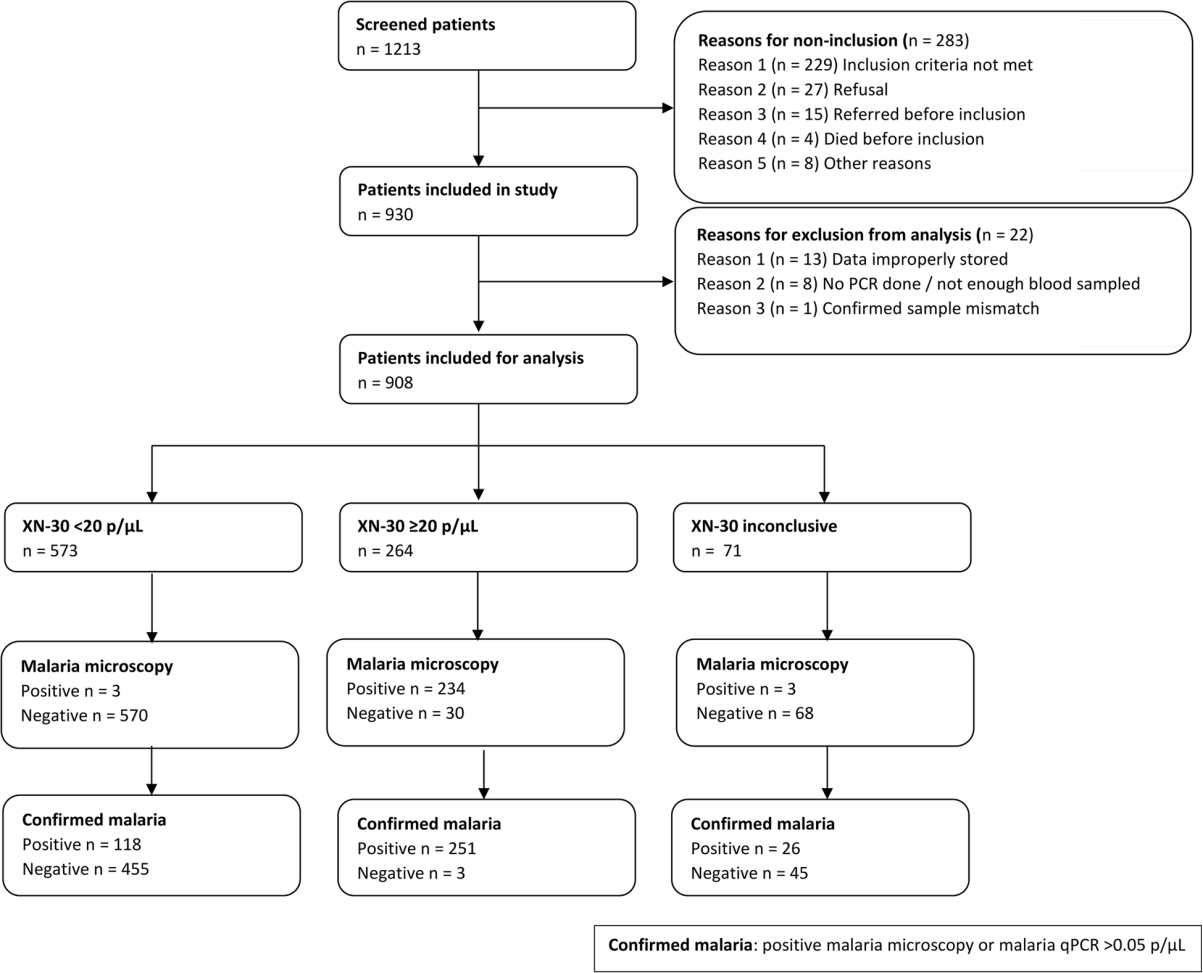
Patient flow of prospective diagnostic validation study in Nanoro, Burkina Faso, excluding patients with incomplete data

We assessed the diagnostic accuracy under field conditions in a malaria hyperendemic region in Burkina Faso. A total of 1213 eligible patients presented at CMA Nanoro, of whom 930 were included (Fig. 4). Fourteen patients were excluded from analysis due to inadequate sample storage or evident sample mismatch. For eight patients, the PCR data was not available due to a shortage of sampled blood. Additionally, in 71 patients (7.8%), the XN-30 scattergram was abnormally distributed and therefore reported as “inconclusive.” There were significantly more patients aged 0–2 and patients with malnutrition among this group. Malaria was significantly less prevalent (Additional file 1: Table S1).

Baseline characteristics for all included patients are presented by age group in Table 1. The percentage of patients who were pre-treated with antimalarials prior to admission ranged between 25.8% (84/335) in adults and 46.1% (83/180) in children 2–5 years old. Severe malaria was detected in 120 (50.0%) patients with microscopically confirmed malaria, of whom 55 (45.6%) fulfilled the criteria of severe anemia. These high numbers are in line with previously reported results [11] and are likely caused by the function of CMA as a district referral hospital for complicated disease. In total, 411 follow-up samples were taken: 59 during hospitalization and 352 at 2-week follow-up.

**Table 1:** Baseline characteristics (*n* = 908)

	Children			Adults
	0–2 years	2–5 years	5–15 years	> 15 years
	*n* = 266	*n* = 180	*n* = 127	*n* = 335
	Median (IQR)	Median (IQR)	Median (IQR)	Median (IQR)
Age distribution (months, years)	12 (8–17) months	36 (28–46) months	8 (6–10) years	41 (29–59) years
Male: female ratio	1.5	1.5	1.3	1.2
Fever (days)	3 (2–3)	3 (2–3)	3 (2–4)	3 (2–4)
Temperature (°C)	38.5 (38.0–39.4)	38.3 (37.9–39.4)	38.2 (37.7–39.0)	38.3 (38.0–39.0)
Systolic BP (mm/Hg)	98 (89–106)	98 (90–108)	102 (95–112)	108 (96–124)
Diastolic BP (mm/Hg)	61 (53–65)	62 (56–69)	63 (59–75)	66 (60–76)
MAP (mm/Hg)	73 (66–79)	75 (67–81)	80 (72–87)	83 (72–92)
Pulse (min)	125 (112–133)	123 (108–135)	116 (100–125)	104 (96–119)
Respiratory rate (min)	35 (32–42)	32 (30–38)	29 (26–30)	27 (26–28)
Hemoglobin (g/dL)	7.8 (5.6–10.0)	8.6 (6.5–10.7)	9.4 (7.8–11.6)	10.3 (8.4–12.8)
WBC (cells × 10^3^/μL)	13.1 (10.0–19.4)	11.2 (7.9–16.5.)	10.6 (7.4–16.6)	7.9 (5.5–12.8)
Platelets (cells × 10^3^/μL)	277 (124–452)	232 (126–358)	271 (181–372)	237 (156–337)
	*n* (%)	*n* (%)	*n* (%)	*n* (%)
Antimalarials taken in past 2 weeks	93 (34.2)	83 (46.1)	54 (42.1)	86 (25.8)
HIV (confirmed before inclusion)	1 (0.4)	2 (1.1)	3 (2.3)	11 (3.3)
Malnourished^1^	112 (43.4)	70 (41.2)	47 (41.6)	84 (28.6)
Severe anaemia^2^	87 (32.7)	50 (27.8)	33 (25.8)	72 (27.7)
Severe malaria	56 (21.0)	47 (26.1)	12 (9.3)	5 (1.5)
Severe anemia	28 (50.0)	15 (31.9)	8 (66.7)	4 (80.0)
Cerebral malaria	8 (14.3)	10 (21.3)	3 (25.0)	0
Respiratory distress	2 (3.6)	4 (8.5)	0	0
Parasitemia > 150.000 p/μL	18 (32.1)	18 (38.3)	1 (8.3)	1 (20.0)

*BP* blood pressure, *MAP* mean arterial pressure, *WBC* white blood cells

^1^Based on *Z*-scores for children < 5 and BMI for older patients, data available for respectively 258, 170, 113, and 294 patients per age group

^2^Based on WHO criteria for severe anemia

The results of malaria diagnostics are presented in Table 2. A total of 240 patients (26.4%) had positive microscopy with parasite densities ranging from 24 to 500,000 p/μL (median 15,614; IQR 745–76,901); 374 patients (41.2%) had a positive qPCR. Sub-microscopic parasitemia (positive qPCR with negative microscopy) was detected in 134 patients (14.8%) with densities ranging from 0.05 to 102.8 p/μL (median 0.68; IQR 0.13–3.1). Six microscopy-positive cases were not detected by qPCR. RDTs were positive in 396 patients; in more than half of the cases, both pLDH and HRP-2 were positive. XN-30 recorded 255 cases of malaria with a median parasite density of 12,390 p/μL (IQR 650–88,656).

**Table 2:** Cases of malaria detected per type of diagnostic, for all included patients (*n* = 916)

	Total	Children			Adults
	All ages	0–2 years	2–5 years	5–15 years	> 15 years
	*n* = 908	*n* = 266	*n* = 180	*n* = 127	*n* = 335
	*n* (%)	*n* (%)	*n* (%)	*n* (%)	*n* (%)
Microscopy	240 (26.4)	88 (33.1)	75 (41.7)	36 (28.1)	41 (12.3)
RDT (minimal 1 test positive)	396 (43.6)	131 (49.3)	118 (65.6)	68 (53.1)	79 (23.7)
Both positive	212 (53.3)	86 (65.7)	68 (57.6)	30 (44.1)	28 (35.0)
Only HRP2 positive	175 (44.2)	44 (33.6)	49 (41.5)	35 (52.2)	47 (59.5)
Only pLDH positive	9 (2.3)	1 (0.8)	1 (0.9)	2 (2.9)	5 (6.3)
qPCR^1^	374 (41.2)	118 (44.4)	104 (57.8)	58 (45.3)	94 (28.1)
> 20 p/μL	218 (24.1)	84 (31.6)	75 (41.7)	29 (22.7)	30 (8.9)
> 1 and < 20 p/μL	74 (8.2)	14 (5.3)	12 (6.7)	18 (14.0)	30 (8.9)
> 0.05 and < 1 p/μL	81 (8.9)	20 (7.5)	17 (9.4)	11 (8.6)	33 (9.9)
XN-30^2,3^	255 (28.0)	87 (32.7)	79 (43.9)	39 (30.7)	50 (14.9)

^1^Excluding six false negative PCRs, which were most likely false negative due to sample mix-up

^2^Excluding 71 patients with an abnormal scattergram, three of whom had positive malaria microscopy

^3^There were three false negative cases detected by microscopy but not detected by XN-30

**Fig. 5:**
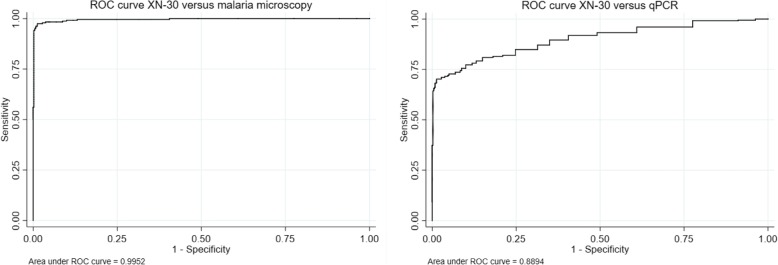
ROC curves XN-30 versus malaria microscopy and qPCR

Tables 3 and 4 present the diagnostic sensitivity, specificity, PPV, and NPV for XN-30 against microscopy and qPCR. Figure 5 shows the corresponding ROC curves at the cutoff value of 20 p/μL . The sensitivity and specificity of XN-30 against microscopy were 98.7% and 96.5%, respectively. In 21 cases, the XN-30 indicated parasitemia whereas microscopy was deemed negative; 19/21 cases were later confirmed as sub-microscopic parasitemia by qPCR. Three samples were false negatives: two cases had low parasite densities (< 20 p/μL in qPCR), the third had a high parasite density and was suspected to be a sample mix-up based on discrepancies in CBC between the XN-30 and a second hematology analyzer used for clinical care. After correction for sub-microscopic malaria, the sensitivity and specificity of the XN-30 increased to 98.8% and 99.7% respectively with a PPV of 99.2% and an NPV of 99.5% (AUC 0.99).

**Table 3:** Diagnostic accuracy of XN-30 compared to malaria microscopy. Data with and without correction for qPCR confirmed sub-microscopic parasitemia cases

	XN-30, uncorrected (*n* = 837)		XN-30, corrected* (*n* = 837)	
	Positive	Negative	Positive	Negative
Positive microscopy	234	3	253	3
Negative microscopy	21	579	2	579
Sensitivity	98.7%		98.8%	
Specificity	96.5%		99.7%	
PPV	91.8%		99.2%	
NPV	99.5%		99.5%	
ROC	0.98		0.99	

*In case of incongruent results between malaria microscopy and XN-30, the microscopy data was adjusted to the PCR result

**Table 4:** Prevalence of disease on qPCR, *N* (%, 95% CI) *McNemar test to compare sensitivity and specificity present. All ratios presented as XN-30 relative to microscopy

	XN-30 and microscopy versus qPCR results (*n* = 837)					
	XN-30		Microscopy		Test ratio (95% CI)	*p* value
	Positive	Negative	Positive	Negative		
qPCR > 0.05 p/μL	253	104	237	120		
qPCR < 0.05 p/μL	2	478	0	480		
Sensitivity	70.9%		66.4%		0.94 (0.90–0.97)	.0009
Specificity	99.6%		100.0%		0	.5
PPV	98.9%		100.0%			
NPV	83.4%		80.0%			
ROC	0.86		0.83			

*qPCR* qualitative polymerase chain reaction, *PPV* positive predictive value, *NPV* negative predictive value

Table 4 presents the diagnostic accuracy for XN-30 and microscopy compared to qPCR. The XN-30 had a significantly higher sensitivity than microscopy to detect qPCR-confirmed parasitemia (70.9% versus 66.4%, *p* = .0009) whereas specificity did not differ between both tests (99.6% versus 100%, *p* = .5). The cutoff value of best fit to qPCR in the current dataset was a parasite density of 26 p/μL, which gave similar results to the proposed cutoff value (sensitivity 69.5%, specificity 98.8%) (Additional file 2: Table S2, Additional file 6: Figure S4).

A secondary objective was to compare the diagnostic performance of XN-30 to diagnose malaria parasitemia against two RDTs. Table 5 shows the diagnostic accuracy of both used RDTs and XN-30 compared to uncorrected microscopy (upper part) and qPCR (lower part). Considering all qPCR-confirmed cases, the sensitivity of XN-30 was significantly higher than pLDH (70.9% versus 60.2%, *p* < .0001), while specificity was equal (both 99.6%, *p* = 1.0). Conversely, XN-30 had higher specificity (99.6% versus 87.7%, *p* < .0001) than HRP-2, while the sensitivity of HRP-2 was higher than that of XN-30 (86.3% versus 70.9%, *p* < .001). The difference in sensitivity between HRP-2 and XN-30 was mostly caused by sub-microscopic parasitemia cases (median parasite density 0.45 p/μL; IQR 0.13–1.8), most of whom (64.0%) had been pre-treated with antimalarials. This suggests that at least part of the cases detected by HRP-2, but missed by XN-30, may have been based on remaining presence of antigens after a recent malaria infection. Among microscopically confirmed cases, XN-30 had an equal or higher sensitivity and higher specificity than HRP-2 and pLDH.

**Table 5:** Prevalence of disease on microscopy (upper part of the table) and qPCR (lower part of the table), *N* (%, 95% CI) *McNemar to compare sensitivity and specificity present ratio of proportions. All ratios presented as XN-30 relative to HRP-2 and pLDH respectively

XN-30 and RDT (HRP-2 and pLDH) versus corrected microscopy results (*n* = 837)										
	XN-30		HRP-2		Test ratio	*p* value	pLDH		Test ratio	*p* value
	Pos	Neg	Pos	Neg	95% CI		Pos	Neg	95% CI	
Microscopy positive	234	3	231	6			208	29		
Microscopy negative	21	579	136	464			9	591		
Sensitivity	98.7%		97.5%		0.99 (0.97–1.01)	*p* = .45	87.8%		0.89 (0.85–0.93)	*p* < .0001
Specificity	96.5%		77.3%		6.47 (4.31–9.73)	*p* < .0001	98.5%		0.43 (0.23–0.81)	*p* = .012
PPV	91.8%		62.9%				95.9%			
NPV	99.5%		98.7%				95.3%			
XN-30 and RDT (HRP-2 and pLDH) versus qPCR results (*n* = 837)										
	XN-30		HRP-2		Test ratio	*p* value	pLDH		Test ratio	*p* value
	Pos	Neg	Pos	Neg	95% CI		Pos	Neg	95% CI	
qPCR > 0.05 p/μL	253	104	308	49			215	142		
qPCR < 0.05 p/μL	2	478	59	421			2	478		
Sensitivity	70.9%		86.3%		1.22 (1.15–1.29)	< 0.0001	60.2%		0.85 (0.80–0.89)	< .0001
Specificity	99.6%		87.7%		0.04 (0.01–0.14)	< 0.0001	99.6%		1.0 (0.07–13.8)	1.0
PPV	99.2%		83.9%				99.1%			
NPV	82.1%		89.6%				77.1%			

*Pos* positive, *neg* negative, *HRP2* histidine-rich protein 2, *pLDH* pan-Plasmodium lactate dehydrogenase, *qPCR* qualitative polymerase chain reaction, *PPV* positive predictive value, *NPV* negative predictive value

**Fig. 6:**
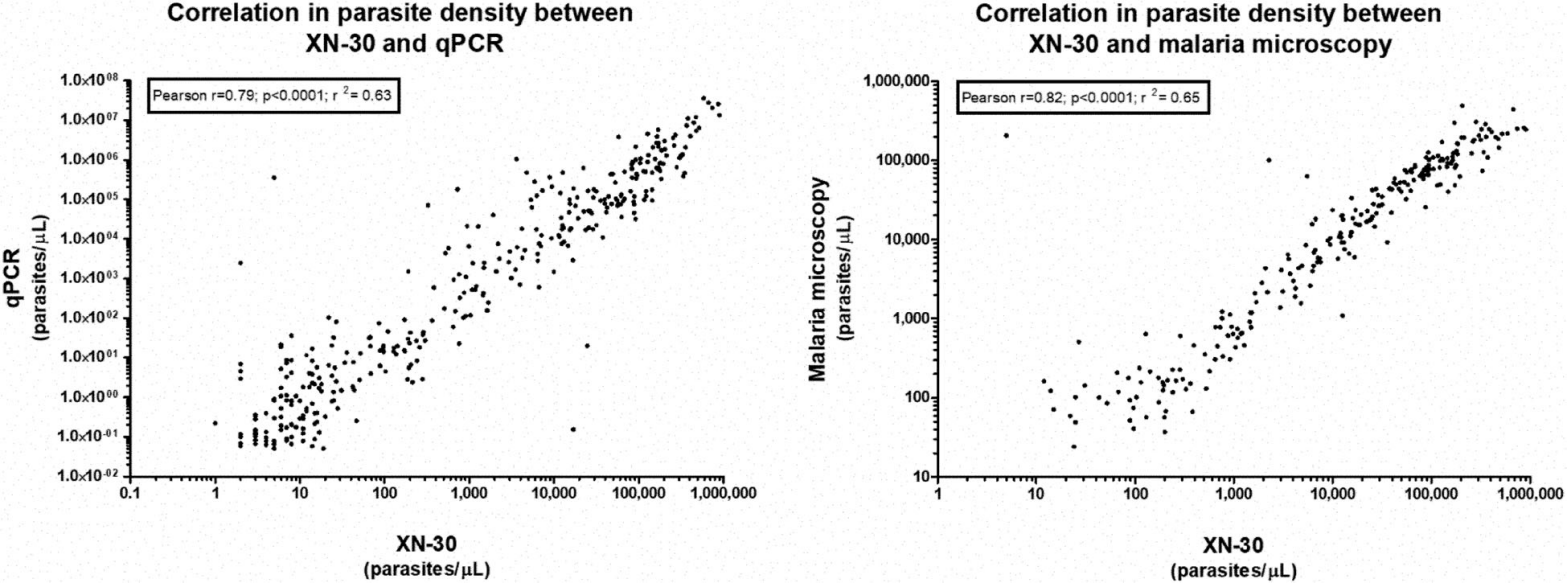
Correlation between parasite density of XN-30 and respectively microscopy and qPCR

We compared parasite densities as recorded by XN-30 with densities quantified by microscopy and qPCR. Figure 6 shows the corresponding correlations (microscopy *r* = 0.82, *p* < .0001; qPCR *r* = 0.79, *p* < .0001). Follow-up samples were available in 125 patients (51.9%) with XN-30-confirmed malaria. The median parasite density dropped from 19,138 p/μL (IQR 1594–98,165) at inclusion to below the limit of positivity (20 p/μL) at follow-up, confirming that XN-30 can be used for treatment follow-up in a clinical setting. A total of 15 cases of non-*falciparum* malaria parasitemia were identified by microscopy, all of which were picked up by XN-30, though they were not differentiated from *P. falciparum.*

## Discussion

This is the first prospective clinical study to present the diagnostic performance of XN-30. The sensitivity of XN-30 to detect *Plasmodium falciparum* parasitemia was superior to that of microscopy in qPCR-confirmed cases, whereas the specificity was comparable. XN-30 had an equal or higher sensitivity than HRP-2 and pLDH respectively, and a higher specificity than both for microscopy-confirmed malaria. Our results are in line with results from a recently published retrospective study on the performance of the XN-30 from South Africa [15]. The accuracy of malaria microscopy (LOD 24 p/μL) in the current study is far above the accuracy of microscopy under field conditions, which is estimated at 50–100 p/μL [16]. The reported accuracy of XN-30 might therefore be an under-estimation compared to most clinical settings. Commonly used RDTs have a detection limit around 100–200 p/μL [17, 18], except when the HRP-2 deletion of *P. falciparum*, as found in Central Africa, is present that prevents the detection of this strain [19].

In case of signs of severe malaria, follow-up of admitted malaria patients, or RDT-confirmed malaria cases with persistent fever despite anti-malarial treatment, the WHO recommends the use of malaria microscopy [20]. Our data suggest that XN-30 could be used as an alternative microscopy in laboratories where hematology analyzers are already used. XN-30 shares many of the advantages of microscopy, including relatively low costs, quantification of parasite density, and monitoring of treatment response. However, in contrast to microscopy, XN-30 can be used by minimally trained staff, without sample preparation, has no observer-dependent viability, and is available around-the-clock, whereas trained malaria microscopists are often limitedly available. Another important additional advantage of the XN-30 is that it simultaneously provides a CBC with each analysis and therefore synchronizes malaria diagnose and management. Severe malaria anemia is a major cause of death, and a delay of blood transfusion is associated with poor outcome [21].

Automated hematology analyzers to diagnose malaria have been studied in the last two decades, applying different techniques such as abnormal depolarization in the granularity of phagocytes, abnormalities in the size of blood cells, or blood cell differentiation patterns [22–27]. However, these methods often carry limitations such as low- and variable sensitivities and specificities due to technical limitations and confounding host- and pathology-specific differences [27, 28]. Direct staining of malaria nucleic acid in parasites and subsequent analysis by a flow cytometry-like technique may prevent drawbacks from indirect tests [29–31]. Several studies have demonstrated that iRBC can be detected through pigmented DNA or RNA strands [32–34]. The XN-30 combines both techniques in a fully automated manner. Since the analyzer can be connected to WiFi, data could be instantaneously linked to any organization involved in epidemiological research and disease control.

The limit of detection as observed in the CHMI study was lower than that of the diagnostic accuracy study (8.1 versus 20p/μL). We have no obvious explanation for this difference; previous studies demonstrate that malaria negative samples with low hemoglobin, high reticulocyte count, thrombocytopenia or hemoglobinopathies had no significant influence on the reliability of the malaria result [8]. Our data as presented in additional Table 1 suggests that this may occur more frequently in very young children and patients with malnutrition, but further research is needed to analyze the origin of this difference.

### Limitations

In the present study, XN-30 had an inconclusive result due to an abnormal scatter gram distribution in 71 cases (7.8%). This implies that when used in clinical settings, approximately one in 12 patients would need an additional malaria diagnostic when the XN-30 is inconclusive. Second, the XN-30 currently does not reliably distinguish *Plasmodium* species prevalent in Burkina Faso. In addition, this study was performed in a malaria hyperendemic setting; therefore, the population-level effect of the sensitivity and specificity could vary by different settings e.g. low-endemicity countries.

## Conclusion

In conclusion, the XN-30 holds promise as a rapid and sensitive test for malaria detection and subsequent treatment monitoring. Since XN-30 provides a CBC with each analysis, it provides critical information for malaria management at a price comparable to CBC. Its higher sensitivity compared to microscopy in low-density parasitemia also makes it a useful tool for mass screening in control programs.

## Additional files

Additional file 1: **Table S1.** Characteristics of patients with inconclusive XN-30 result. (DOCX 18 kb)

Additional file 2: **Table S2.** ROC analysis of cutoff value of best fit, XN-30 compared to qPCR. (DOCX 20 kb)

Additional file 3: **Figure S1.** CHMI study participant inclusion flow. (TIF 73 kb)

Additional file 4: **Figure S2.** Correlation of the XN-30 with microscopy and qPCR, in cultured parasites. (TIF 660 kb)

Additional file 5: **Figure S3.** Stability data of cultured ring-stage samples. (TIF 181 kb)

Additional file 6: **Figure S4.** ROC curve at cutoff value of best fit, XN-30 compared to qPCR. (TIF 1006 kb)

## Abbreviations

AUC: Area under the curve
CBC: Complete blood count
CHMI: Controlled human malaria infection
CMA: Centre Medicale avec Antenne Chirurgicale (District Hospital of Nanoro)
CRF: Case report form
CRUN: Clinical Research Unit of Nanoro
DNA: Deoxyribonucleic acid
EDTA: Ethylenediaminetetraacetic acid
GCLP: Good clinical and laboratory practices
HRP2: Histidine-rich protein-2
IMS: Infection Manager System
iRBC: Infected red blood cells
LOD: Limit of detection
MI-RBC(#): Outcome measure of XN-30: absolute parasite density expressed as parasites/μL
MI-RBC(%): Outcome measure of XN-30: parasites as a percentage of infected red blood cells
N: Absolute number
NPV: Negative predictive value
p/μL: Parasites/microliter
pLDH: pan-*Plasmodium* species parasite-lactate dehydrogenase
PPV: Positive predictive value
qPCR: Quantitative polymerase chain reaction
RBC: Red blood cells
RDT: Rapid diagnostic test
RNA: Ribonucleic acid
ROC: Receiver operation characteristics curve
WBC: White blood cells
WHO: World Health Organization
