# Supplementary material for: The XN-30 hematology analyzer for rapid sensitive detection of malaria: a diagnostic accuracy study
Source: BMC Med. 2019 May 31;17:103. doi: 10.1186/s12916-019-1334-5 (PMC6543632; doi:10.1186/s12916-019-1334-5)
Supplement: Supplementary file 1 — Table S1. Characteristics of patients with inconclusive XN-30 result. (DOCX 18 kb) [file 12916_2019_1334_MOESM1_ESM.docx]

**S1 Table.** Characteristics of patients with inconclusive XN-30 result (n=71) and reliable XN-30 result (n=837) included in the analyses. Percentages are column percentages. Significance calculated by multiple variable regression analysis, correcting for malnutrition and age.

|  | Inconclusive  XN-30 result | conclusive  XN-30 result | p-value |
| --- | --- | --- | --- |
|  | n=71 | n=837 |  |
|  | *n (%)* | *n (%)* | *p-value* |
| Age distribution |  |  |  |
| Patients 0-2 years | 37 (52.1) | 229 (27.4) | <.0001 |
| Patients 2-5 years | 9 (12.7) | 171 (20.4) | .12 |
| Patients 5-15 years | 5 (7.0) | 122 (14.6) | .08 |
| Patients ≥15 years | 20 (28.2) | 315 (37.6) | .13 |
| Sex |  |  |  |
| Male | 48 (67.6) | 475 (56.7) | .08 |
| Female | 23 (32.4) | 362 (43.3) | .08 |
| Antimalarials in past 2 weeks | 26 (36.7) | 279 (33.3) | .57 |
| Malnourished | 36 (52.3) | 277 (36.1) | .003 |
| Severe anaemia | 25 (35.2) | 216 (25.8) | .09 |
|  |  |  |  |
|  | *Median (IQR)* | *Median (IQR)* | *p-value* |
| Fever (days) | 3 (2-4) | 2 (2-3) | .23 |
| Temperature (°C) | 38.5 (38.0-39.4) | 38.3 (38.0-39.1) | .52 |
| Systolic BP (mm/Hg) | 101 (90-110) | 101 (93-112) | .79 |
| Diastolic BP (mm/Hg) | 63 (57-70) | 62 (56-72) | .46 |
| Pulse (min) | 124 (101-132) | 116 (100-128) | .82 |
| Respiratory rate (min) | 32 (28-36) | 30 (27-34) | .92 |
| Hemoglobin (g/dl) | 9.0 (5.0-10.7) | 9.8 (7.2-11.4) | .28 |
| WBC (cells x10^3^/µL) | 13.4 (6.8-19.4) | 10.6 (7.0-15.6) | .75 |
| Platelets (cells x10^3^/µL) | 240 (113-425) | 249 (149-370) | .88 |
|  |  |  |  |
|  | *n (%)* | *n (%)* | *p-value* |
| Malaria smear | 3 (4.2) | 237 (28.3) | <.0001 |
| RDT (minimal 1 test positive) | 21 (29.6) | 375 (44.8) | .0001 |
| *Both positive* | 3 (14.3) | 209 (55.7) | <.0001 |
| *HRP-2 positive* | 17 (80.9) | 158 (42.1) | .29 |
| *pLDH positive* | 1 (4.8) | 8 (2.1) | .71 |
| qPCR > 0.05 p/µL | 23 (32.4) | 357 (42.7) | .093 |
| *qPCR > 20 p/µL* | 7 (30.4) | 212 (59.4) | .003 |
| *qPCR >1 and <20 p/µL* | 8 (34.8) | 66 (18.5) | .32 |
| *qPCR >0.05 and <1 p/µL* | 8 (34.8) | 73 (20.4) | .47 |
|  |  |  |  |
