# Supplementary material for: The XN-30 hematology analyzer for rapid sensitive detection of malaria: a diagnostic accuracy study
Source: BMC Med. 2019 May 31;17:103. doi: 10.1186/s12916-019-1334-5 (PMC6543632; doi:10.1186/s12916-019-1334-5)
Supplement: Supplementary file 2 — Table S2. ROC analysis of cutoff value of best fit, XN-30 compared to qPCR. (DOCX 20 kb) [file 12916_2019_1334_MOESM2_ESM.docx]

**S2 Table. ROC analysis and cut-off value for best fit between XN30 and qPCR, with corresponding sensitivity and specificity.**

| **Cut-off value Parasite density**  **XN-30 (p/µl)** | | **Sensitivity**  **(%)** | **Specificity**  **(%)** | **Correctly classified**  **(%)** |
| --- | --- | --- | --- | --- |
| > 0 | | 100 | 0 | 42.7 |
| > 1 | | 99.4 | 3.8 | 44.6 |
| > 2 | | 88.2 | 8.9 | 47.4 |
| > 3 | | 96.1 | 22.5 | 53.9 |
| > 4 | | 93.3 | 39.2 | 62.3 |
| > 5 | | 91.9 | 51.0 | 68.5 |
| > 6 | | 89.6 | 59.6 | 72.4 |
| > 7 | | 87.1 | 65.0 | 74.4 |
| > 8 | | 84.9 | 68.5 | 75.5 |
| > 9 | | 82.1 | 75.2 | 78.1 |
| > 10 | | 81.5 | 78.9 | 80.1 |
| > 11 | | 80.9 | 81.9 | 81.5 |
| > 12 | | 79.3 | 85.0 | 82.6 |
| > 13 | | 78.2 | 86.9 | 83.2 |
| > 14 | | 77.3 | 88.1 | 83.5 |
| > 15 | | 75.6 | 90.0 | 83.9 |
| > 16 | | 74.5 | 91.3 | 84.1 |
| > 17 | | 73.7 | 91.7 | 83.9 |
| > 18 | | 72.8 | 93.1 | 84.5 |
| > 19 | | 72.3 | 95.0 | 85.3 |
| > 20 | | 71.9 | 95.2 | 85.4 |
| > 21 | | 71.7 | 95.4 | 85.4 |
| > 22 | | 71.7 | 95.6 | 85.4 |
| > 23 | | 71.2 | 96.3 | 85.5 |
| > 24 | | 71.2 | 96.5 | 85.7 |
| > 25 | | 70.3 | 97.3 | 85.8 |
| **> 26** | | **69.5** | **98.8** | **86.3** |
| > 27 | | 69.2 | 98.8 | 86.1 |
| > 28 | | 68.6 | 98.8 | 85.9 |
| > 29 | | 68.4 | 98.8 | 85.8 |
| > 30 | | 68.4 | 98.9 | 85.9 |
| > 31 | | 68.4 | 99.2 | 86.0 |
| > 34 | | 68.1 | 99.2 | 85.9 |
| > 36 | | 67.5 | 99.2 | 85.8 |
| > 42 | | 67.5 | 99.2 | 85.7 |
| > 43 | | 66.9 | 99.2 | 85.4 |
| > 44 | | 66.7 | 99.2 | 85.3 |
| > 47 | | 66.4 | 99.2 | 95.2 |
| > 51 | | 66.1 | 99.2 | 85.0 |
|  | |  |  |  |
| **AUC** | **Standard error** | **95% CI** |  |  |
| 0.89 | 0.013 | 0.87-0.91 |  |  |
