# Supplementary figures and images for: The XN-30 hematology analyzer for rapid sensitive detection of malaria: a diagnostic accuracy study
Source: BMC Med. 2019 May 31;17:103. doi: 10.1186/s12916-019-1334-5 (PMC6543632; doi:10.1186/s12916-019-1334-5)

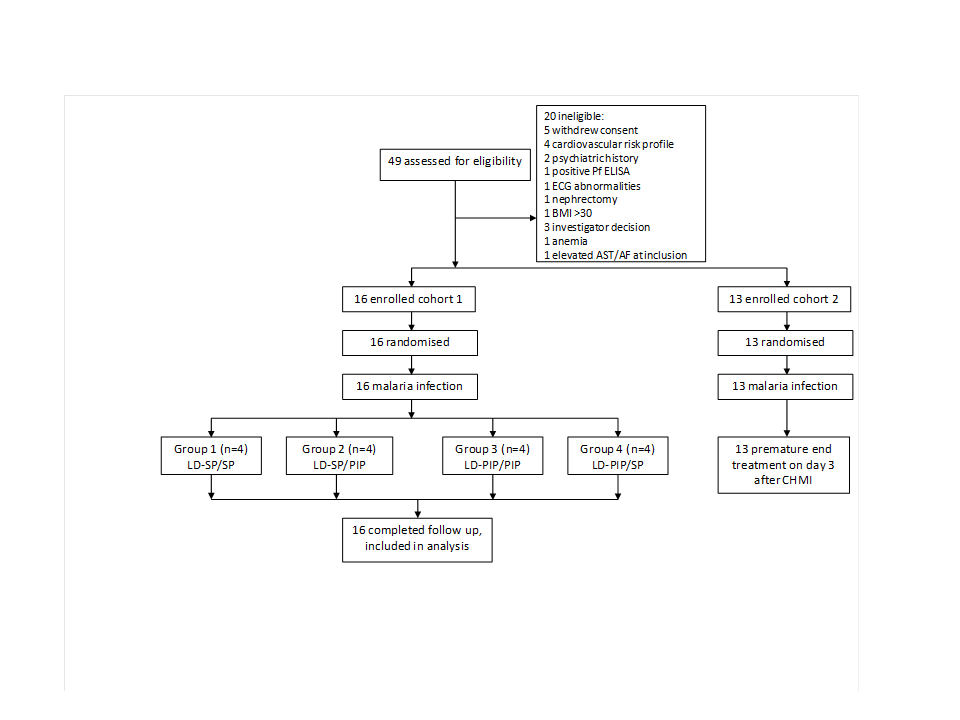

Supplement: Supplementary file 3 — Figure S1. CHMI study participant inclusion flow. (TIF 73 kb) [file 12916_2019_1334_MOESM3_ESM.tif]

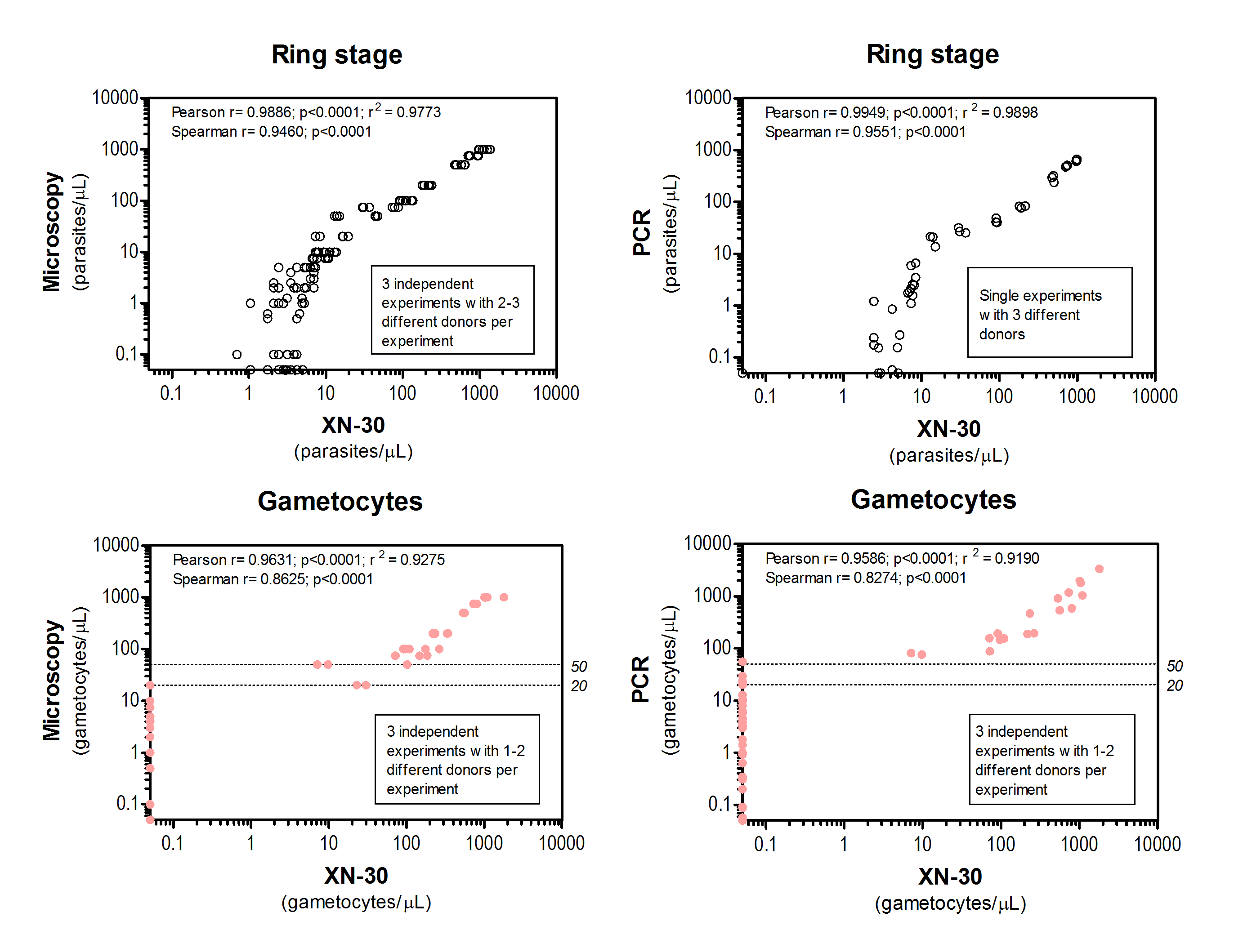

Supplement: Supplementary file 4 — Figure S2. Correlation of the XN-30 with microscopy and qPCR, in cultured parasites. (TIF 660 kb) [file 12916_2019_1334_MOESM4_ESM.tif]

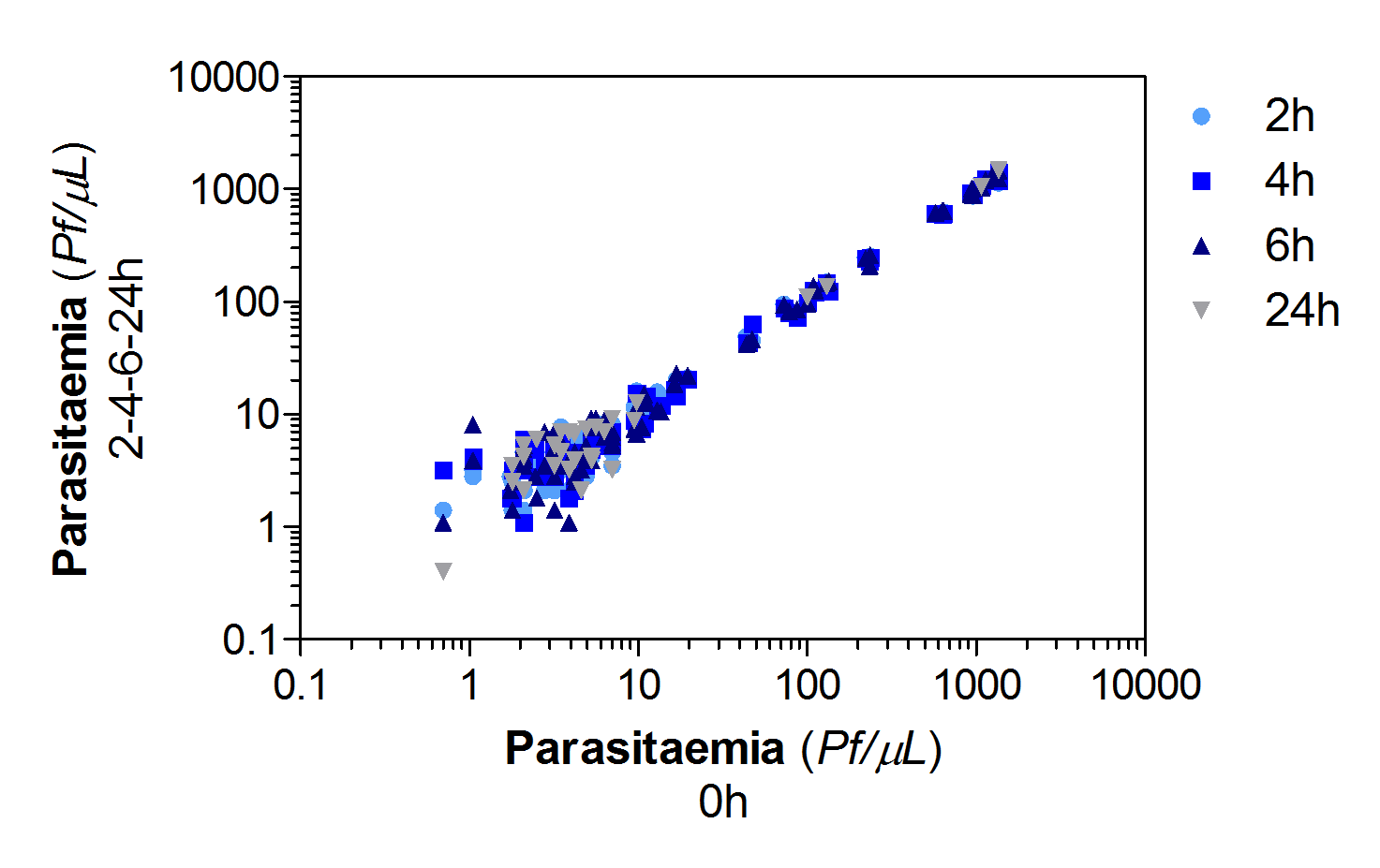

Supplement: Supplementary file 5 — Figure S3. Stability data of cultured ring-stage samples. (TIF 181 kb) [file 12916_2019_1334_MOESM5_ESM.tif]

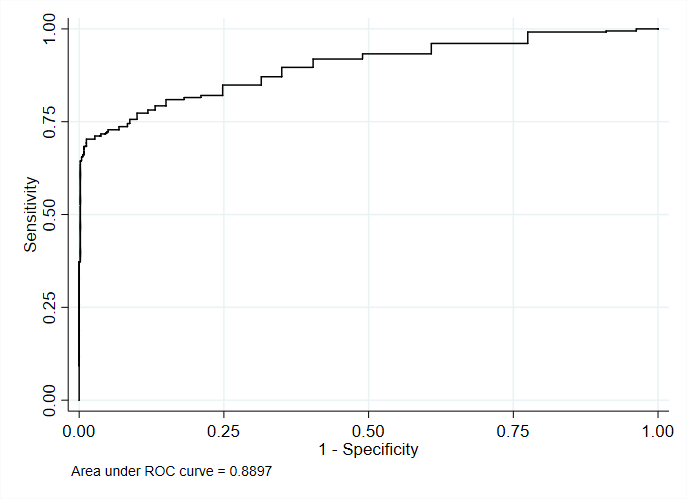

Supplement: Supplementary file 6 — Figure S4. ROC curve at cutoff value of best fit, XN-30 compared to qPCR. (TIF 1006 kb) [file 12916_2019_1334_MOESM6_ESM.tif]
